# Valorization of tea waste: Composition, bioactivity, extraction methods, and utilization

**DOI:** 10.1002/fsn3.4011

**Published:** 2024-03-04

**Authors:** Tümay Gözdem Çakmak, Beyza Saricaoglu, Gulay Ozkan, Merve Tomas, Esra Capanoglu

**Affiliations:** ^1^ Department of Food Engineering, Faculty of Chemical and Metallurgical Engineering Istanbul Technical University Istanbul Turkey; ^2^ Department of Food Engineering, Faculty of Engineering and Natural Sciences Istanbul Sabahattin Zaim University Istanbul Turkey

**Keywords:** extraction of bioactives, phenolic compounds, spent tea leaves, waste valorization

## Abstract

Tea is the most consumed beverage worldwide and has many health effects. Although there are many different types of tea, black tea and green tea comprise 98% of total tea production in the world. Tea waste production consists of withering, crushing, fermentation, drying and finally packaging processes. All of the waste generated during this production line is called tea waste. Tea production results in a significant amount of waste that cannot be effectively used for value creation. This waste contains many different components including protein, fiber, caffeine, and polyphenols. Due to its rich composition, it can be revalorized for different purposes. In this study, the general composition and bioactive compounds of tea waste were reviewed. Despite the fact that there have been few studies on the bioactivity of tea waste, those studies have also been discussed. The extraction techniques that are used to separate the compounds in the waste are also covered. It has been indicated that these valuable compounds, which can be separated from tea wastes by extraction methods, have the potential to be used for different purposes, such as biogas production, functional foods, food additives, silages, soluble packaging materials, and adsorbents. Although there are some studies on the revalorization of tea waste, new studies on the extraction of bioactive compounds are necessary to improve its utilization potential.

## Graphical abstract

Tea is consumed in high quantities and therefore a large amount of waste is generated. Tea waste contains important food components such as phenolic compounds, protein and fiber. For this reason, tea waste has the potential to be utilized in many areas such as functional food production, biodegradable packaging production, absorbent and silage production.

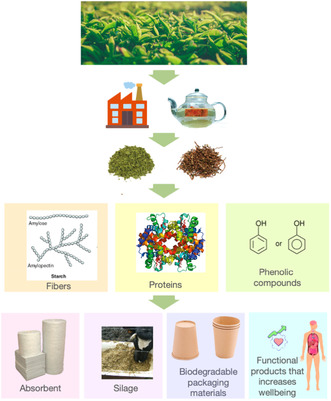

## 1 INTRODUCTION

The tea plant is an evergreen species of the genus *Camelia* with dark green leaves and white flowers. It is a plant native to China. The two most commonly used species in the production of different types of tea are *Camelia sinensis* and *Camelia assamica*. Tea prepared by pouring hot water on processed tea leaves is a type of beverage consumed daily around the world. In addition to its taste and aroma, it is a valuable beverage with cultural value and health benefits (Ho et al., 2018; Kosińska & Andlauer, 2014). It is a widely consumed beverage around the world. According to the estimates, 78% of the world's tea production includes black tea production and 20% green tea production. The production of other types of tea is only 2%. In addition to black tea consumed all over the world, an increase in green tea consumption has been observed with the emergence of positive effects on health. Oolong tea and green tea are tea varieties native to Asia in general. Pu‐erh and white tea varieties are generally preferred in Asia (Kosińska & Andlauer, 2014).

Tea is a healthy beverage and the consumption demand for tea is increasing day by day. Thus, tea production and accordingly the amount of tea waste are increasing. Tea wastes occur during the pruning of tea trees, after daily tea consumption, and during industrial production. Tea waste is generated in every process applied to tea in factory production. These processes can be counted as withering, crushing, fermentation, drying and finally packaging processes. According to Negi et al. (2022), approximately 90% of tea leaves become tea waste after processing and consumption. For example, it is known that as a result of 857,000 tons of tea produced in India, 190,000 tons of tea waste is generated. According to Hussain et al. (2018), approximately 30,000 tons of tea waste are disposed of on the Black Sea coast of Turkey. If such high amounts of tea waste are not utilized, the economic burden will increase, and it will cause serious environmental problems. For this reason, healthy bioactive components, such as polyphenols in the tea residue, should be separated by various methods. It is possible to separate these healthy bioactive compounds with extraction methods. Thus, these wastes are converted into biomass and used in different areas, and recycled. As a result of the many research, thanks to the rich content of tea waste, its use in various fields such as bioenergy production, as an adsorbent, in addition to silages, as food additives, used in functional foods has been tried to be widespread giving hope for the future.

This study aims to better recognize tea, which is a widely consumed beverage worldwide, to emphasize and encourage the importance of utilizing tea waste by reviewing the valuable compounds contained in tea waste and different applications for the evaluation of these compounds.

## 2 PROCESSING OF TEA AND GENERATION OF TEA WASTE

**FIGURE 1:**
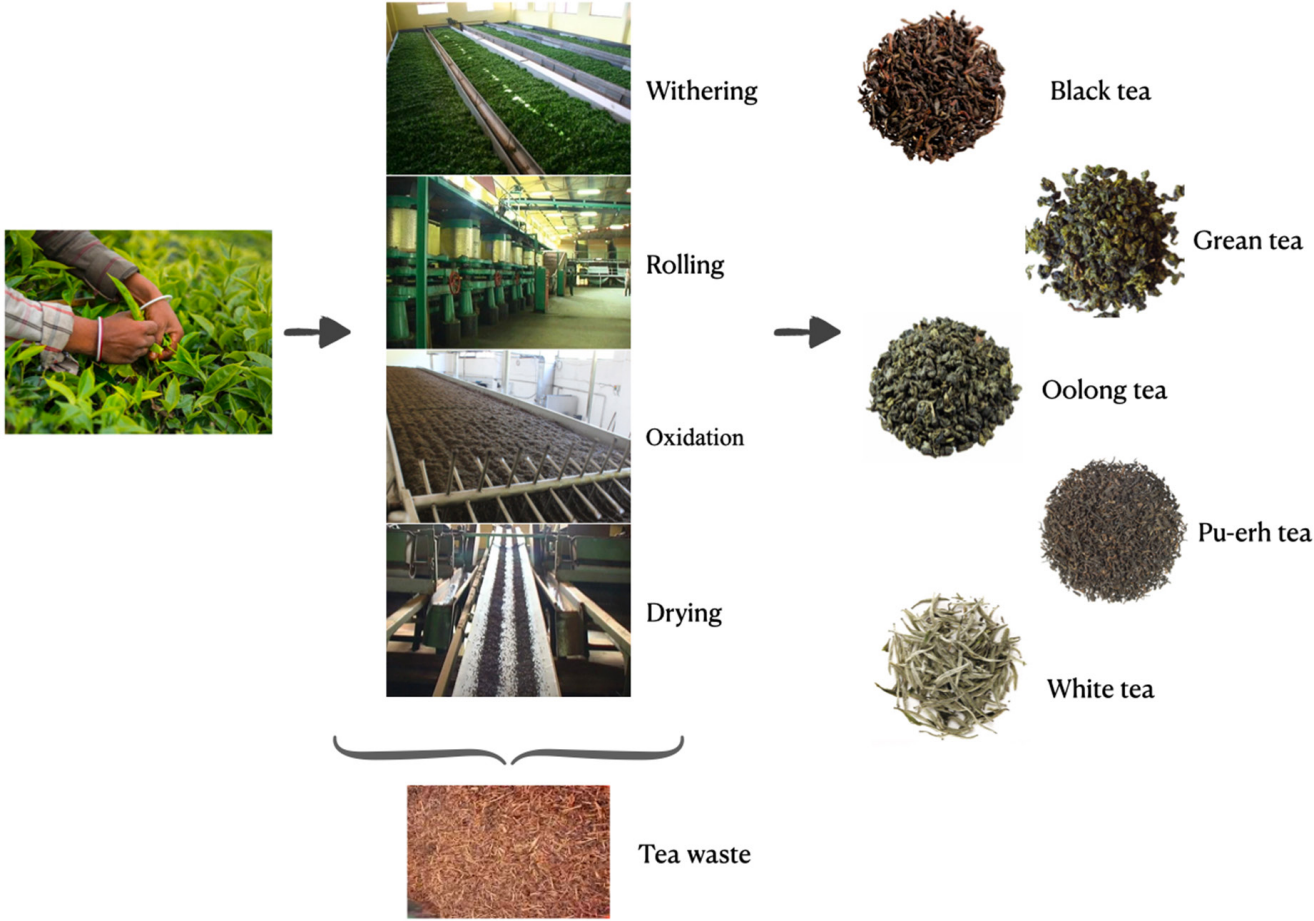
Processing of tea and generation of tea waste.

Although tea was traditionally collected by hand for many years, it is now frequently collected by leaf‐cutting scissors or plucking machines. After the collection of tea, different processes, such as oxidation and fermentation, are applied to obtain the final product. There are several types of tea, including black tea, green tea, white tea, oolong tea, and Pu‐erh tea (Figure 1). The reason for this diversity in teas is due to the difference in the oxidation and fermentation stages that occur during tea processing. While the tea plant of the genus *C*. *assamica* is used in the production of black tea and Pu‐erh tea, the leaves of the genus *C*. *sinensis* are used in the production of green tea (Ho et al., 2018; Kosińska & Andlauer, 2014).

### 2.1 Black tea production

Black tea production generally consists of four stages, including withering, rolling, enzymatic oxidation, and drying (Ho et al., 2018; Tosun & Karadeniz, 2005). Viable and wilted leaves are crushed to oxidize tea catechins through peroxidase and polyphenol oxidase enzymes. Characteristic flavor formation occurs as a result of the conversion of peroxidase and polyphenol oxidase enzymes during black tea production. The catechin content is reduced as a result of this enzymatic oxidation. In this way, the bitterness and astringency in the taste of tea are reduced. Fermentation is generally carried out at 40°C for 1–2 h. In the final stage of black tea production, the crushed leaves are heated and dried. Thus, enzymes are inactivated (Ho et al., 2018; Tanaka & Matsuo, 2020).

### 2.2 Green tea production

Green tea is a type of nonfermented tea, such as white tea. In the production of green tea, all oxidation enzymes, including polyphenol oxidase, are inactivated. For this purpose, fresh tea leaves are withered immediately after harvest by applying high temperature, stir‐frying, or steam shock. Peroxidase and polyphenol oxidase enzymes are inactivated by pan‐frying or steaming the tea leaves. This ensures that the compositions of fresh leaves and green tea polyphenols are similar (Ho et al., 2018;Tanaka & Matsuo, 2020; Tosun & Karadeniz, 2005). The next steps after withering the leaves in green tea production are rolling and drying (Tanaka & Matsuo, 2020; Tosun & Karadeniz, 2005).

### 2.3 Oolong tea production

For the production of oolong tea, the leaf margins are often partially crushed to separate the catechins and enzymes. Next, the fermentation step of oolong tea takes place semi‐enzymatically (Ho et al., 2018). Generally, in the production of oolong tea, a slight withering process is applied to the fresh leaves by applying a hot air flow or with the help of the sun, so that the water of the leaves is partially removed and cooled with the help of airflow. The leaves are then curled, a process to increase the contact surface for the fermentation step, also known as oxidation. Semi‐fermentation process is applied in the production of oolong tea. After fermentation, another important step, drying, is applied. It is important to repeat the drying process at regular intervals because, under different storage conditions, tea can continue enzymatic oxidation by absorbing moisture from the environment (Koca & Bostancı, 2014; Tosun & Karadeniz, 2005). In oolong tea production, the oxidation stage is kept shorter than the black tea production (Salman & Özdemir, 2018).

### 2.4 Pu‐erh tea production

Pu‐erh tea is a rare variety of tea that belongs to the category of Chinese black tea (Ho et al., 2018). Generally, one bud and 2–3 leaves of fresh and large green tea leaves are used in the production of Pu‐erh tea. Fresh tea leaves are left on bamboo mats for 8 h to dry, which ensures partial drying. Then, the leaves are heated in the drum to inactivate the polyphenol oxidase enzyme, but the enzyme activity is partially preserved (Lv et al., 2013). A milder heating process is used than green tea, so not all the enzymes are inactivated (Ho et al., 2018).

The rolling process applied to Pu‐erh tea takes less time than green tea. This results in curled leaves in a loose state. This is a situation that facilitates the postfermentation process. The rolled tea leaves are dried with the help of the sun at a temperature of more than 30°C. At the end of this drying process, approximately 8% moisture content is obtained. After fermentation, the sun‐dried leaves undergo some oxidation and decomposition, resulting in the characteristic sensory properties of Pu‐erh tea. Autoclaving and second drying processes are applied to Pu‐erh tea after the final fermentation stage. Thus, it can be compressed in different ways (Lv et al., 2013). Various microorganisms, such as *Aspergillus niger*, *Saccharomyces*, *Penicillium*, and *Aspergillus candidus*, play a role in the quality of Pu‐erh tea including aroma, color, taste, and health effects (Ho et al., 2018; Lv et al., 2013).

### 2.5 White tea production

The tips of *Camelia sinensis* tea buds have white and hair‐like structures at harvest time. White tea took its name from these white structures (Ho et al., 2018). Only *Camellia sinensis* (L.) O. Kuntze plant can be used in the production of white tea. Although it is used in a very simple process, it is a very expensive type of tea since the buds can only be picked by hand and it involves the use of very fresh tea buds. The collected buds are generally dried in the shade, under the sun, or in tumble dryers. Depending on which method will be used, the buds are first wilted and then dried. They are left to dry for 23–34 h at room temperature and in the shade until the moisture content reaches around 5%–7%. Harvesting the raw material in the first shoot period is a very important factor in the quality of white tea. Leaves should be collected and processed at the right time, taking into account the climatic conditions (Salman & Özdemir, 2018).

### 2.6 Generation of tea waste

Tea is one of the most consumed nonalcoholic beverages and its consumption has increased considerably in recent years around the world. The total tea production in the world is about 6 million tons, and its global consumption is about 5.8 million tons. During the production of tea, tea waste is generated and it mostly consists of fibrous waste. A small part of this waste is used as raw material for feedstock or as compost. However, a very large part of the waste generated during tea processing is thrown away. Incorrectly incinerated or disposed tea waste causes major environmental problems. These wastes pollute the water, soil, and air. Therefore, sustainability studies have increased in recent years. The concept of “waste to wealth,” which is an environmentally friendly concept, is growing. This concept helps to reduce wastewater treatment costs, and supports sustainability. It aids the valorization of modified and raw tea wastes (Debnath et al., 2021).

On the other hand, tea waste is also generated after the tea is brewed and called as “Spent tea leaves (STLs)”. Tea leaves are processed by extraction also known as brewing and used in various industrial products. After brewing, the residue (STLs) correspond to app. 90% (Hussain et al., 2018), and this large amount of solid waste produced in different points including houses, tea shops, hotels, etc. causes environmental pollution. Disposal of these wastes requires economic support, large areas, and efforts. Due to this increasing requirement and demand, many studies have been carried out by scientists to find solutions for the valorization of this waste (Negi et al., 2022; Sermyagina et al., 2021).

This waste is the unused portion of tea that has been processed in tea production. Therefore, it has a similar composition to processed tea. Although there are many different types of tea, tea waste with a similar content is obtained from all of them. The various tea production methods and the extraction of tea waste are summarized in Figure 1. Tea waste has a very rich content, and in addition to its nutritional components such as fiber and protein, it also contains functional components, such as phenols, tannins, steroids, and saponins. Especially some types are accepted as a good source of proteins and have a variety of amino acids (Jiang et al., 2019; Kondo et al., 2018; Xu et al., 2021). It is known that green tea leaf waste is superior to black tea leaf waste in terms of chemical components. Although black tea leaf waste is richer in organic matter and dry matter, its crude protein ratio is lower than the green tea waste (Negi et al., 2022).

During the processing of green tea leaves, drying is applied immediately after harvest, which has the function of stopping oxidation and enzymatic activity. Thus, the harmful effects of polyphenols and proteins are prevented (Kondo et al., 2018). The processing of black tea leaves is aimed at accelerating the oxidation, thus enzymes, such as peptidase and polyphenol oxidase, are also activated. Black tea leaf silage has more fiber content than green tea leaf silage. Tea waste has been mostly used in fiber‐rich food products; green tea leaf silage, on the other hand, has been also used in functional foods due to its rich phenolic and lactic acid contents. In addition, tea waste is an important source of saturated fatty acids (Fadhil & Saeed, 2016).

Tea waste has significant structural and nutritional potential, and it can be used in the production of both food and nonfood products. By contrast, raw compost is often dumped in landfills and its use is very limited. Recently, due to increasing environmental and nutritional concerns, these wastes have been evaluated as biocomponents (Negi et al., 2022).

## 3 COMPOSITION AND BIOACTIVE PROPERTIES OF TEA WASTE

### 3.1 Composition of tea waste

Even though there are many different types of tea, the composition of tea waste is mostly similar. The main components of tea waste are structural proteins, lignin, cellulose, hemicellulose, secondary metabolites, and minerals (Guo et al., 2021; Panneerselvam et al., 2011). There are only few studies in which the proximate composition of tea waste was analyzed. Zheng et al. (2017) reported that the proximate composition of green tea on a dry basis was 27.2% crude protein, 2.11% lipid, 5.1% ash, and 8.0% moisture. Also, they reported that green tea waste contains 6.1% polyphenols, 1.14% free amino acids, and 14.1% crude fiber (Zheng et al., 2017). Another study reported that tea waste contains 20.82% crude protein, 5.23% lipid, 4.92% ash, %13.69 moisture, 17.06% crude fiber, 4.58% cellulose, 13.96% hemicellulose, 0.54% calcium, 0.36% phosphorus, and 0.18% silica (Sudheer Babu et al., 2022).

### 3.2 Phenolic compounds in tea waste

The phenolic content of the tea plant may be related to the maturity of the leaf. The type of tea plant, climate, season, and growing conditions are other factors that can affect the polyphenolic profile (Ahmed et al., 2019). A study showed that mature leaves contain higher levels of epigallocatechin (EGC) and epicatechin (EC), whereas, young leaves and buds contain epicatechin gallate (ECG) and epigallocatechin gallate (EGCG). In addition, it has been observed that mature leaves contain less caffeine than younger leaves. This difference was attributed to the collection method and collection period (Ho et al., 2018). Theaflavins, which are oxidized dimers of catechins, and thearubigins, which are oxidized polymers of catechins, are compounds that give black tea its characteristic properties. These are the features that determine the quality of the tea, such as the mouth feeling of the tea and its dark reddish color. In addition, thearubigins make up 60%–70% of the dissolved solids in the infusion. Therefore, thearubigins are very important components of black tea (Ho et al., 2018).

Tea waste, on the other hand, has a similar phenolic composition to processed tea. Studies that have reported the phenolic composition of tea waste are summarized in Table 1. Uğurlu and Güçlü Üstündağ (2017) reported that the phenolic profile of green tea waste contains catechin (C), epicatechin, epigallocatechin gallate, epicatechin gallate, epigallocatechin, gallocatechin (GC), and gallocatechin gallate (GCG). In another study, Serdar et al. (2017) reported that black tea contains catechin, epicatechin, epigallocatechin, epigallocatechin gallate, and gallic acid (GA). In another study performed by Güçlü Üstündağ et al. (2016) and Güçlü Üstündağ et al. (2016), epicatechin, epigallocatechin gallate, gallocatechin gallate, epicatechin gallate, and gallic acid were detected in the black tea waste. Similarly, Balcı‐Torun et al. (2021) reported that black tea waste contains epicatechin, epigallocatechin, epigallocatechin gallate, epicatechin gallate, catechin, catechin gallate (CG), gallocatechin, and gallic acid.

**TABLE 1:** Phenolic compounds in tea waste.^a^

Sample	C	EC	GC	EGC	ECG	EGCG	CG	GCG	GA	Reference
Black tea waste	0.80 ± 0.00	4.10 ± 0.04	0.60 ± 0.01	1.20 ± 0.01	0.70 ± 0.00	1.60 ± 0.01	0.60 ± 0.03	−	0.70 ± 0.00	(Balcı‐Torun et al., 2021)
Black tea waste	+	+	−	+	−	+	−	−	+	(Serdar et al., 2017)
Black tea waste	−	4.69 ± 0.09	−	−	0.26 ± 0.02	1.05 ± 0.05	−	−	0.65 ± 0.02	(Güçlü Üstündağ et al., 2016)
Green tea waste	0.90 ± 0.06	10.56 ± 1.04	1.54 ± 0.10	21.66 ± 3.70	6.06 ± 1.39	45.58 ± 10.14	−	1.34 ± 0.64	−	(Uğurlu & Güçlü Üstündağ, 2017)

Abbreviations: −, not detected or analyzed; +, detected but not quantified; C, Catechin; CG, Catechin gallate; EC, Epicatechin; ECG, Epicatechin gallate; EGC, Epigallocatechin; EGCG, Epigallocatechin gallate; GA, Gallic acid; GC, Gallocatechin; GCG, Gallocatechin gallate.

^a^ Results are presented as milligrams/gram (mg/g) sample.

### 3.3 Bioactive properties of tea waste

Tea waste with its high bioactive content exhibits various bioactive properties. For instance, the dietary fiber in the tea waste is rich in bound polyphenols leading to good antihyperglycemic effects (Huang et al., 2022). There is a synergistic effect between dietary fibers and bound polyphenols, and helps reducing the harmful bacteria in the gut and increasing the beneficial bacteria. Thanks to the hypoglycemic and antioxidant properties of tea residue proteins, they can be used in the preparation of peptides that provide natural blood pressure‐lowering effects (Xingfei et al., 2020).

The phenolic compounds in the tea waste, such as catechin and its derivatives, have various bioactive properties, including anticancer, antioxidant, and anti‐inflammatory activities (dos Santos et al., 2020). Studies indicated that tea waste, similar to that of tea itself, exhibits antioxidant activity. For instance, the antioxidant activity of green tea and black tea wastes was measured and the half‐maximum inhibitory concentrations (IC_50_) of samples were reported as 57.5 and 88.9 μg/mL, respectively (Martono, 2010). As reported in the study, although green tea waste (13.18 gallic acid equivalent [GAE] mg/g sample) had lower total phenolic content than black tea waste (35.77 GAE mg/g sample), green tea waste exhibited high antioxidant capacity. This is presumed to be due to the difference in the phenolic profile since some phenolics exhibit higher antioxidant activity than the others. Studies focusing on the bioactive properties of tea waste are summarized in Table 2.

**TABLE 2:** Bioactive properties of tea waste.

Sample	Component	Main bioactivity	Outcome	Reference
Black tea oven waste	Phenolic compounds	Antioxidant activity	3.59 ± 0.38, 0.92 ± 0.11, and 2.38 ± 0.25 μmol TE/mg extract antioxidant activity for DPPH, FRAP, and ABTS, respectively, with the 50% ethanolic extract	Güçlü Üstündağ et al. (2016)
Black tea oven waste	Phenolic compounds	Antimicrobial activity	3.62 ± 0.50, 3.89 ± 0.72, and 3.90 ± 0.68 zone of inhibition (mm) against *S*. *aureus*, *S*. *flexneri*, and *B*. *cereus*, respectively, with the 80% ethanolic extract	Güçlü Üstündağ et al. (2016)
Tea waste	Phenolic compounds	Antioxidant activity	More than 80% DPPH free and hydroxyl radicals scavenging activity; High survival rate on zebrafish embryos	Gao et al. (2020)
Black tea waste	Ethanolic extract	Antioxidant activity	No significant difference between the antioxidant activities of raw and spent tea leaves	Abdeltaif et al. (2018)
Black tea waste	Methanolic extract	Antioxidant activity	More than 40% of DPPH radical scavenging activity for 80% ethanolic extract	
Black tea waste	Phenolic compounds	Antioxidant activity	App. 2.2 μmol TE/mg extract antioxidant activity with ABTS test for 80% ethanolic extract	Otağ, (2023)
Black tea waste	Phenolic compounds	Antimicrobial activity	25.00 ± 1.41, 22.00 ± 0.70, 20.00 ± 1.41, and 16.00 ± 0.70 zone of inhibition (mm) against *E*. *coli*, *S*. *aureus*, *B*. *cereus*, and *S*. *typhimurium*, respectively, with 40 μL of extract	Otağ, (2023)
Black tea waste	Tea extract	Antioxidant activity	Highest extraction yield observed at the highest concentration; Higher antioxidant activity with ethyl acetate compared to hot water and methanol extraction	Farhoosh et al. (2007)
Green, oolong, and black tea infusion waste		Antioxidant activity, antihypertensive activity	The highest antioxidant activity in green tea waste (18.76 ± 0.46 μg/mL IC_50_ for DPPH method, 25.41 ± 0.21 μg/mL IC_50_ for ferric ion reducing power, and 27.57 ± 0.42 μg/mL IC_50_ hydroxyl radical scavenging activity); The highest ACE inhibitory effect (46.72 μg/mL. IC_50_) in black tea waste	Xue et al. (2018)
Green tea waste	Phenolic compounds	Antioxidant activity	57.48 ± 0.33 mg Trolox/g extract at 2.5% (g/mL) by DPPH method	Makalesi et al. (2020)
Tea waste	Water extract of bioactive components	Antioxidant activity	App. 5.5 FRAP value at a concentration of 10.0 mg/mL extract; More than 90% DPPH free radical scavenging activity at a concentration of 10.0 mg/mL extract; More than 70% hydroxyl radical scavenging activity at a concentration of 10.0 mg/mL extract; More than 70% superoxide anion radical scavenging activity at a concentration of 10.0 mg/mL extract	Sui et al. (2019)
Black tea waste	Catechins	Antioxidant activity	12.17 ± 0.00 mM Trolox/mg compound activity with CUPRAC method; 3.42 ± 0.09 μg/mL SC_50_ value with DPPH method; 2.78 ± 0.05 μg/mL SC_50_ value with ABTS method	Kantar et al. (2022)

In addition to the studies related to enrichment of foods for human consumption, tea waste has been studied to be used as feed for animals such as goats and sheeps. Tea waste addition to the feed of animals resulted with an improvement in rumen fermentation (Kondo et al., 2018) and lactic acid bacteria growth (Kondo, Naoki, et al., 2004).

## 4 EXTRACTION METHODS OF BIOACTIVE COMPOUNDS FROM TEA WASTES

Due to the tea waste being rich in bioactive components, various extraction methods are applied to extract bioactives from tea waste. There are four main active components in tea waste, including polyphenols, protein, fiber, and caffeine (Miao et al., 2022). Different extraction methods can be used for different active components found in tea waste which is expected to be environmentally friendly and economic. The energy spent and the time required during extraction are the factors which are especially important during the processing of large amounts of waste (Serdar et al., 2017).

### 4.1 Extraction of phenolic compounds from tea waste

Different methods are used to extract the polyphenols present in tea waste including solvent extraction, enzyme‐assisted extraction, ultrasound‐assisted extraction, microwave‐assisted extraction, high‐pressure processing extraction, supercritical liquid extraction, subcritical solvent extraction, subcritical water extraction, and chromatography (Miao et al., 2022; Mushtaq et al., 2017).

#### 4.1.1 Solvent extraction

Solvent extraction is a method mostly used to extract caffeine from tea waste. In this method, caffeine in the tea waste is denatured and then it is extracted with alkali or lime. The solvent is removed by evaporation. The remaining residue is extracted with hot water for purification and crystallization (Miao et al., 2022). As another method, it is possible to extract denatured tea waste with hot water. After this step, the caffeine in the tea waste is transferred to organic solvents by using dichloromethane or ethyl acetate. Caffeine is then obtained through purification and crystallization steps (Miao et al., 2022; Serdar et al., 2017).

#### 4.1.2 Enzyme‐assisted extraction

Some of the bioactive components in plant materials are bound to carbohydrates and therefore it is difficult to extract them in the aqueous phase. Enzyme‐assisted extraction provides the liberation of bound compounds and as a result, compounds are easily transferred to the extraction solvent (Nadar et al., 2018). Enzyme‐assisted extraction provides higher extraction yield by lowering the damage of bioactives caused by the extraction method. On the other hand, it can be expensive and there is still a biological residue that needs to be removed after extraction. There are many carbohydrates in tea waste that can be catalyzed and easily removed by enzyme‐assisted extraction. In a study, an enzyme‐assisted extraction process was applied to extract the polyphenols in black tea wastes. Tea wastes were hydrolyzed with various enzymes and supercritical carbon dioxide and ethanol (SC‐CO_2_ + EtOH) were used as cosolvents. Conventional solvent extraction was also used for comparison. For the conventional method, the ratio of EtOH + H_2_O (80:20, v/v) was used. As a result of the study, it was reported that the use of Kemzyme^®^ increased the extraction efficiency by 5 times (Mushtaq et al., 2017). Kemzyme^®^ is a multiple enzyme product that contains enzymes, such as lipase, protease, α‐amylase, pectinase, and hemicellulase. It is used to break down nonstarch polysaccharides (Abbas et al., 1998). The conditions of the study were determined as a temperature of 45°C, the hydrolysis time was 98 min, the pH was 5.4, and the tea waste was treated with 2.9% (w/w) Kemzyme^®^. It has been concluded that the enzyme‐assisted SC‐CO_2_ + EtOH extraction method is an environmentally friendly and effective method for the recovery of polyphenols in tea waste with antioxidant activity (Mushtaq et al., 2017).

#### 4.1.3 Microwave‐assisted extraction

Microwave‐assisted extraction is based on the ability of polar chemicals to absorb microwave energy in conjunction with their dielectric constant. In contrast to traditional heating, microwave radiation heats a sample uniformly, rapidly, and simultaneously throughout. The moisture within cells allows microwave radiation to heat them, and when the moisture is evaporated, pressure is applied to the cell wall. The high pressure breaks down the cell wall, allowing the components to pass into the solvent (Marić et al., 2018). In the microwave heating method, the effect of temperature was investigated without using any organic solvents and catalysts. It was reported that the content of phenolic compounds in tea wastes increased when the temperature was increased from 110°C to 230°C (Miao et al., 2022). Extraction using hot water is a simple method, but it is insufficient when recycling large amounts of waste is desired because this method requires high levels of energy and long extraction times. The microwave‐assisted extraction method is considered as a good approach due to its low‐energy consumption and shorter extraction time required. With the power of the microwave, the structure of the cell wall is disrupted and thus, catechins and caffeine can more easily pass into the extraction solvent. In a study, microwave‐assisted extraction was applied at 80–100°C for 30 min with a solvent:tea ratio of 20:1 (mL/g) and compared to the conventional method. Results showed that both extraction methods had similar yields. However, authors suggested microwave‐assisted extraction as a more effective method since it was more economical and took shorter time to apply (Serdar et al., 2017).

#### 4.1.4 Supercritical fluid extraction

Supercritical fluid has liquid‐like density and solubility due to its gas‐like diffusion, viscosity, and surface tension properties. Supercritical fluids can easily diffuse into solid materials due to their low viscosity and relatively high diffusion properties. An increase in the diffusion results in an increase in the extraction yield. Moreover, generally recognized as safe (GRAS) solvents are used in supercritical fluid extraction (da Silva et al., 2016). In particular, carbon dioxide is one of the most frequently used solvents for supercritical fluid extraction and it has many advantages, including being safe for humans and the environment, use of lower temperatures (around 30°C) for the extraction of bioactive molecules, and protection of extracts from air and light during the extraction procedure (Barbosa et al., 2014). These properties enable the compounds to be extracted more efficiently. In a study, polyphenols in tea wastes were extracted by taking advantage of the high solubility of supercritical CO_2_. The supercritical extraction fluid method was used for the sequential extraction of catechins and caffeine from green tea waste. The study was carried out at four different temperatures (30, 40, 50, and 60°C), times (1, 2, 3, and 5 h), and pressure values (10, 20, 25, and 30 MPa), and it was observed that extraction at 60°C and 25 MPa pressure for 3 h provided the optimum conditions for the extraction yield of catechin. However, it has been stated that it is not a very suitable method for caffeine extraction from tea waste (Sökmen et al., 2018).

#### 4.1.5 Subcritical solvent extraction

Another method that can be used to separate tea polyphenols from tea waste is semicontinuous subcritical solvent extraction. The polarity of the solvent decreases under subcritical conditions and in this way, the diffusion properties of solvent improve. Combining the subcritical water with an organic solvent improves extraction yield, extraction time, solvent consumption, and solubility of compounds (Kwon & Chung, 2015). In a study, it was observed that this method increased the solubility of polyphenols in black tea waste more than the hot water extraction and the yield of polyphenols was increased (Miao et al., 2022). In a study, optimum conditions for extraction of phenolics from black tea waste by subcritical solvent extraction were analyzed. Results showed that the optimum conditions were a 1:1 ethanol–water solvent ratio, 0.3 MPa pressure, and 125°C temperature. It was also mentioned that, this method is suitable to extract nonextractable polyphenols in tea waste (Rajapaksha & Shimizu, 2022).

#### 4.1.6 Subcritical water extraction

Since water is a polar molecule, it is not an effective solvent for extracting nonpolar or organic molecules. However, the diffusion properties of water can be enhanced by changing the pressure (1–22.1 MPa) and temperature (100–374°C). Subcritical water has lower viscosity, dielectric constant, and surface tension, therefore it can act like organic solvents and dissolve some compounds that have low and medium polarity (Zhang et al., 2020). Subcritical water extraction is a method used to remove caffeine from tea waste. It is known that caffeine recovery is higher in this method compared to the traditional hot water extraction method. While the recovery rate in conventional hot water extraction is 0.46%, it reaches 0.77% in subcritical water extraction. This value is valid under conditions of 0.5 mm average particle size, 175°C temperature, and 4 g/min water flow rate in subcritical water extraction (Miao et al., 2022). A study observed that 77% of the caffeine in the tea wastes was recovered within approximately 1.5 h, and no relation between the extraction speed and extraction efficiency and the pressure was observed (Shalmashi et al., 2010). In another study, optimum conditions (temperature, time, and flow rate) to extract L‐theanine and catechins from green tea waste using subcritical water extraction were analyzed. Maximum yield for L‐theanine was observed at 90°C in 60 min (2 mL/min) as 4.38 ± 0.48 mg/g dry wt. The extraction yield of catechin was temperature‐ and time‐dependent. The maximum yield for catechin was observed at 90°C and 60 min at 2 mL/min and 110°C and 45 min at 4 mL/min (Uğurlu & Güçlü Üstündağ, 2017).

#### 4.1.7 Chromatography

One of the methods that can be used to separate tea polyphenols from tea waste is chromatography. The method is mostly applied after an extraction procedure to separate the phenolic fractions and purification of phenolic groups or compounds. In a study, column chromatography was used to separate and purify polyphenols from decaffeinated green tea waste. After the purification by chromatography, the purity of green tea polyphenols was found to be approximately 98%, and the catechin content was more than 91%. In this method, the first step is to remove the caffeine and the second step is to separate polyphenols. Different percentages of ethanol were used for these steps. It has been observed that the recovery of tea polyphenols could be as high as 86%. It has many advantages such as being simple, economical, highly efficient, clean, environmentally friendly, and toxic residue‐free method having good stability (Miao et al., 2022). Column chromatography is a method used to separate and ensure purification of the bioactive compounds. In another study, gas chromatography method was applied to ensure the recovery of phenolic compounds from tea wastes. As a result of the study, it was observed that the extraction of phenols was quite high with a yield of 95.02%. It also provides an environmentally friendly method for the recovery of tea waste (Tahir et al., 2021).

### 4.2 Extraction of protein compounds from tea waste

Tea waste could also be a valuable source of proteins for which different extraction methods have been developed. This is very important in terms of recycling tea residues to obtain high‐value products. The protein extraction methods applied to tea waste are enzymatic methods and alkaline extraction methods (Miao et al., 2022).

#### 4.2.1 Alkaline extraction

The alkaline method is widely used for the extraction of proteins present in tea waste. Most of the proteins found in tea waste are soluble at alkaline pH but insoluble at neutral pH. This is due to the disulfide bonds they contain and their hydrophobic properties. In this method, the extraction rate generally varies between 15% and 60%. To increase the extraction rate of the proteins, the temperature may be kept as high as 60–95°C. Although the use of high temperature increases the extraction rate, it can cause the proteins to denature. As a result, protein bioactivity and functionality may be affected, and toxic compounds may form. In cases where the alkali concentration is high, Maillard reactions increase and cause excessive browning. Despite these disadvantages, alkaline extraction method can be still preferred because it is suitable for industrial production and this method can be combined with other methods to prevent these problems (Miao et al., 2022).

#### 4.2.2 Enzymatic extraction

One of the methods used for the extraction of tea waste proteins is the enzymatic extraction method. In this method, protease is used as an enzyme which hydrolyzes the tea residue protein, facilitating the extraction. It also has effects such as shortening the peptide chain and reducing the molecular weight. In a study, the extraction time was kept as 1.5 h, the temperature was 60°C, the latitude was 2.5%, the pH was 9.5, and the liquid–solid ratio was 20:1, and it was determined that these conditions provided the optimum conditions for extraction. The extraction rate obtained under these conditions was 56.95% (Yuan et al., 2013).

### 4.3 Extraction of fiber compounds from tea waste

Another compound found in tea waste is natural dietary fiber. The tea waste mostly contains insoluble dietary fiber. Some studies focused on the modification of insoluble dietary fiber to soluble dietary fiber since soluble fiber has many health benefits such as controlling blood sugar and reducing lipid metabolism in the human body. Several methods including acid‐base method, fermentation and enzyme treatment are used to modify the fiber in tea waste and extract the soluble dietary fibers (Miao et al., 2022). Dietary fiber in tea waste is usually extracted using the enzymatic extraction method. For this purpose, starch is catalyzed with α‐amylase, and then the protein is catalyzed with papain. Dietary fiber is separated via centrifugation of flocculation depending on its solubility (Chen et al., 2020; Huang et al., 2021).

## 5 UTILIZATION OF TEA WASTES FOR DIFFERENT PURPOSES

Recycling of agricultural waste is vital for sustainability. In this regard, studies on how to recycle tea waste will provide great benefits for countries with high tea production and tea consumption. Tea wastes used for high‐value applications have a great potential to be used in more valuable applications rather than other agricultural waste materials that are recycled. It has been reported that there are many usage areas of tea residues, including bioenergy, functional foods, food additives, packaging materials, silage and adsorbents.

### 5.1 Functional foods

Recently, there has been a high interest in providing health benefits with food consumption. Providing health effects to foodstuffs from waste sources is also an important research area. Since tea waste contains various bioactive compounds that have different physiological functions and are beneficial for human health, it has the potential to be used in the production of functional foods. For instance, tea fruit peel, an agricultural waste of the tea production industry, contains phenols with high levels of antioxidants. Besides, green tea fruit peel extract has antiangiogenesis and anti‐obesity effects. Therefore, the obesity prevention effect of green tea fruit peel extract can be used in the formulation of functional foods (Chaudhary et al., 2014).

Some studies evaluate the usage of tea wastes in food products. For instance, melon seed powder and tea stalk caffeine were used for the production of functional energy drinks. As a result of the study, the beverage that has been formulated with tea stalk caffeine showed a better antimicrobial property against some pathogen microorganisms, including *Staphylococcus aureus*, *Escherichia coli*, *Bacillus cereus*, and *Aspergillus niger*. In addition to this, sensory properties, such as color, mouthfeel, odor, taste, and total acceptance, were improved. The beverage was more nutritious with improved vitamin and mineral contents as well as total phenolic compounds (1.56 mg gallic acid equivalents/mg) and antioxidant capacity. It was concluded that the use of this combination provides cheaper food ingredients in the practical application of the production of functional energy drinks (Selahvarzi et al., 2021).

### 5.2 Nutritional additives

Separation of active components from tea wastes by extraction is another recovery method to use them as food additives (Sui et al., 2019). It is known that tea waste can also be used as a natural colorant. Tea contains some compounds that give its unique color. Of these, thearubigins are responsible for their reddish‐brown color, theaflavins for their yellowish‐brown color, pheophorbide for their brownish color, carotene for their yellow color, and flavonol glycosides for their light yellow color (Roofigari Haghighat, et al., 2020).

Due to the high phenolic levels of tea, natural pigments obtained by the extraction method are observed to have antioxidant activity (Miao et al., 2022). In a study, the most suitable extraction conditions were investigated to separate natural pigments from black tea wastes. As a result, optimum processing conditions were determined as 80°C by using ethanol:water as solvent with a ratio of 50:50 (Roofigari Haghighat, et al., 2020). In another study, a colorless jelly (aloe vera) was colored using brown commercial color pigment purchased from the local market or brown colorant extracted from tea waste. By adding 10 mL of colorant extracted from the tea waste and 1 mL of the commercial colorant, the same color of the jelly was obtained (Roofigari Haghighat, et al., 2020). As a result of the study, it was observed that there was no difference in the taste of the jelly colored separately with natural and commercial pigments, and even the jellies colored with natural pigments were preferred (Miao et al., 2022). In another study, it was reported that the shelf life of the food could be extended, and the quality of the food could be preserved by the application of polyphenols in the tea wastes as a natural antioxidant source. Because, the reactions that allow the proliferation of free radicals that occur during the oxidation process can be inhibited by polyphenols (Abdeltaif et al., 2018).

### 5.3 Biodegradable packaging materials

Green packaging is a material that prevents the formation of environmental pollution by biodegrading in nature. There are several methods to use tea wastes in the production of green packaging systems. Some of these methods are formation of nanocrystalline cellulose (NCC) and microcrystalline cellulose (MCC). Nanocellulose is a biomass material obtained by decomposing natural cellulose through biological, chemical, and physical means and is available at the nanoscale (Miao et al., 2022). In a study, acid hydrolysis was applied to tea wastes to prepare NCC. As a result of the study, it was observed that the NCC efficiency reached 49.87% (Guo et al., 2020). Due to its flexibility and high strength against shrinkage, NCC can be used as a packaging substrate in green packaging production. In a study where oolong tea was used as a raw material, MCC was prepared by applying acid hydrolysis (Zhao et al., 2018). In addition to these outcomes, it was obtained that NCC and MCCs prepared using wood showed weaker thermal stability than NCC and MCCs prepared using tea wastes.

### 5.4 Silage

It is also possible to use tea waste in animal feed since tea leaves contain many active ingredients, such as proteins, polyphenols, and minerals, and it has been observed that some insoluble active ingredients are abundant in the tea residues left behind after the extraction process. Tea waste as a ruminant feed is, therefore, a good supplement because it reinforces protein, minerals, fiber, and secondary metabolites (Miao et al., 2022). Kondo et al. (2006) studied the effects of adding tea waste to silage on animals. As a result of the addition of green tea waste to the silage, the fermentation efficiency was found to be affected positively for the growth of lactic acid bacteria. In another study with goats, the effect of green tea waste addition on the nutritional value of silage was investigated. For this purpose, green tea waste, from a local beverage company, was added to whole grain oat silage and stored in silos for 50 days. As a result, the addition of green tea waste increased the lactic acid concentrations after fermentation. Furthermore, dense tannin and crude protein contents in the silage were found to be higher with this treatment (Kondo, Kita, & Yokota, 2004).

### 5.5 Adsorbent

After some biotransformation, waste tea leaves can be used as a low‐cost adsorbent in the removal of ions, such as Cd, Cr (IV), Hg, and As (V) (Negi et al., 2022). Tea residue has a porous and loose structure. It also contains components, such as carboxylic acid, aromatic, phenolic, hydroxyl and oxyl groups as well as cellulose and hemicellulose. These components act as a low‐cost adsorbent that removes metal ions and harmful small substances from wastewater. Biosorption is one of the methods used to remove heavy metal ions and, when compared to other traditional methods, it is more efficient and less costly. It is known that tea wastes can be used as an adsorbent to remove Ni^2+^ and Zn^2+^ from water (Miao et al., 2022).

Industrial wastewater contains compounds called hydralazine hydrochlorides. These compounds may cause symptoms, such as increased heart rate, nausea, and vomiting. Some studies have been carried out to remove these pollutants from water. For this purpose, tea wastes were used as adsorbents. Endocrine‐disrupting (EDC) substance is also a very important pollutant that can be found in wastewater, surface waters, and landfill leachate. The use of tea wastes in the adsorption of harmful components of EDC in wastewater provided positive results, in which the adsorption capacity was found to be the highest between acidic and neutral pH (Ifelebuegu et al.,&amp;#x000A0;^2015^).

Tea waste has a high added value and it is necessary to establish a successive industrial system for the recycling of tea waste. Various opportunities could be offered to increase other potential uses of tea waste. With the progresses in the technology, it is possible that tea wastes can be used in many fields, such as biomedicine, food, agriculture, materials, and electrochemistry, and innovative studies should be encouraged for the implementation and improvement of these applications.

## 6 SAFETY LEGISLATION AND CHALLENGES

One of the most important criteria for the valorization of food waste is the safety aspect of the waste. Countries have developed regulations to ensure food safety for the use of food wastes. Codex Alimentarius and the European Community (Regulation No. 178/2002, Article 2) regulate the legislation regarding the use of food waste as food ingredients or additives in Europe. In the USA, the Food and Drug Administration (FDA) is responsible for the legislation of food waste. In particular, Federal Food, Drug, and Cosmetic Act (1938) (latest amendment in 2018) and the Public Health Service Act (PHSA 42 US C) are used for this purpose (Vilas‐Boas et al., 2021).

The wastes to be evaluated should not possess any physical, chemical, or biological risk for the consumers. For instance, compounds such as heavy metals, pesticides, microorganisms, chemical residues such as solvents, and toxins that may be found in food waste are hazardous to human health (Costa et al., 2019). Moreover, tea and tea waste may contain some chemical residues due to factors such as fertilizers used during the cultivation of tea and potential pollution in irrigation resources. Therefore, tea waste must be microbially safe during storage and must be kept in a way that does not cause the formation of any microbial toxins. It is possible to prevent safety problems by using good agricultural and production practices and effective storage of tea waste.

## 7 CONCLUSION

In this review, production, generation, bioactive properties, and utilization of tea wastes were discussed. Tea is a beverage that is consumed in high quantities all around the world which leads to the generation of large amounts of tea waste. Tea waste contains bioactive compounds that have many health benefits, in particular, antioxidant activity. Valorization of these compounds has the potential to contribute significantly to the economy and environmental protection of the countries in which high amounts of tea are produced. By applying different extraction methods, polyphenols, proteins, caffeine, and fibers in tea waste can be separated, which are important for nutritional value and biological activity aspects. However, studies on the extraction of bioactive compounds in tea waste are still limited. For this reason, alternative methods for extraction of bioactive compounds can be developed and an effective system at industrial scale should be established. Besides, extraction processes should be optimized leading to the valorization of these high‐value‐added components, thus providing advantages to save energy and money and protect the environment. The utilization of tea waste either directly or after extraction of the bioactive compounds, and their incorporation in different functional food systems together with other application alternatives should be encouraged.

## AUTHOR CONTRIBUTIONS

**Tümay Gözdem Çakmak:** Investigation (equal); writing – original draft (lead). **Beyza Saricaoglu:** Conceptualization (equal); writing – original draft (supporting). **Gulay Ozkan:** Conceptualization (equal); writing – review and editing (equal). **Merve Tomas:** Writing – review and editing (equal). **Esra Capanoglu:** Conceptualization (equal); supervision (equal); writing – review and editing (equal).

## FUNDING INFORMATION

This research did not receive any specific grant from funding agencies in the public, commercial, or not‐for‐profit sectors.

## CONFLICT OF INTEREST STATEMENT

The authors have no conflicts of interest to declare for this study.

## ETHICS STATEMENT

The current study is a review article and did not involve direct experimentation on any animals and humans and no ethical approval was required.

## Data Availability

All data that support the findings of this study are included in this review article.

## References

[fsn34011-bib-0062] Abbas, W. , Khan, S. H. , & Sarwar, M. (1998). Sunflower oil meal as a substitute for soybean meal in broiler rations with or without multienzyme (Kemzyme). Pakistan Veterinary Journal, 18, 124–129.

[fsn34011-bib-0001] Abdeltaif, S. A. , Sirelkhatim, K. A. , & Hassan, A. B. (2018). Estimation of phenolic and flavonoid compounds and antioxidant activity of spent coffee and black tea (processing) waste for potential recovery and reuse in Sudan. Recycling, 3(2), 27. 10.3390/RECYCLING3020027

[fsn34011-bib-0061] Ahmed, S. , Griffin, T. S. , Kraner, D. , Schaffner, M. K. , Sharma, D. , Hazel, M. , Leitch, A. R. , Orians, C. M. , Han, W. , Stepp, J. R. , Robbat, A. , Matyas, C. , Long, C. , Xue, D. , Houser, R. F. , & Cash, S. B. (2019). Environmental factors variably impact tea secondary metabolites in the context of climate change. Frontiers in Plant Science, 10, 939. 10.3389/fpls.2019.00939 31475018 PMC6702324

[fsn34011-bib-0002] Balcı‐Torun, F. , Sultan Özdemir, K. , LastNameMavuş, R. , & Torun, M. (2021). Siyah çay üretim atıklarından konsantre çay ekstraktı üretiminde krema oluşum koşullarının ve bileşiminin belirlenmesi. Gıda the Journal of Food, 46(2), 339–350. 10.15237/gida.GD20145

[fsn34011-bib-0003] Barbosa, H. M. A. , De Melo, M. M. R. , Coimbra, M. A. , Passos, C. P. , & Silva, C. M. (2014). Optimization of the supercritical fluid coextraction of oil and diterpenes from spent coffee grounds using experimental design and response surface methodology. The Journal of Supercritical Fluids, 85, 165–172. 10.1016/J.SUPFLU.2013.11.011

[fsn34011-bib-0004] Chaudhary, N. , Bhardwaj, J. , Seo, H. J. , Kim, M. Y. , Shin, T. S. , & Kim, J. D. (2014). Camellia sinensis fruit peel extract inhibits angiogenesis and ameliorates obesity induced by high‐fat diet in rats. Journal of Functional Foods, 7(1), 479–486. 10.1016/J.JFF.2014.01.008

[fsn34011-bib-0005] Chen, J. , Huang, H. , Chen, Y. , Xie, J. , Song, Y. , Chang, X. , Liu, S. , Wang, Z. , Hu, X. , & Yu, Q. (2020). Effects of fermentation on the structural characteristics and in vitro binding capacity of soluble dietary fiber from tea residues. LWT, 131, 109818. 10.1016/J.LWT.2020.109818

[fsn34011-bib-0006] Costa, J. G. , Vidovic, B. , Saraiva, N. , do Céu Costa, M. , Del Favero, G. , Marko, D. , Oliveira, N. G. , & Fernandes, A. S. (2019). Contaminants: A dark side of food supplements? Free Radical Research, 53(sup1), 1113–1135. 10.1080/10715762.2019.1636045 31500469

[fsn34011-bib-0007] da Silva, R. P. F. F. , Rocha‐Santos, T. A. P. , & Duarte, A. C. (2016). Supercritical fluid extraction of bioactive compounds. TRAC Trends in Analytical Chemistry, 76, 40–51. 10.1016/J.TRAC.2015.11.013

[fsn34011-bib-0008] Debnath, B. , Haldar, D. , & Purkait, M. K. (2021). Potential and sustainable utilization of tea waste: A review on present status and future trends. Journal of Environmental Chemical Engineering, 9(5), 106179. 10.1016/J.JECE.2021.106179

[fsn34011-bib-0009] dos Santos, A. N. , de L Nascimento, T. R. , Gondim, B. L. C. , Velo, M. M. A. C. , de A Rêgo, R. I. , do C. Neto, J. R. , Machado, J. R. , da Silva, M. V. , de Araújo, H. W. C. , Fonseca, M. G. , & Castellano, L. R. C. (2020). Catechins as model bioactive compounds for biomedical applications. Current Pharmaceutical Design, 26(33), 4032–4047. 10.2174/1381612826666200603124418 32493187

[fsn34011-bib-0010] Fadhil, A. B. , & Saeed, L. I. (2016). Sulfonated tea waste: A low‐cost adsorbent for purification of biodiesel. International Journal of Green Energy, 13(1), 110–118. 10.1080/15435075.2014.896801

[fsn34011-bib-0011] Farhoosh, R. , Golmovahhed, G. A. , & Khodaparast, M. H. H. (2007). Antioxidant activity of various extracts of old tea leaves and black tea wastes (*Camellia sinensis* L.). Food Chemistry, 100(1), 231–236. 10.1016/J.FOODCHEM.2005.09.046

[fsn34011-bib-0012] Gao, T. , Shi, Y. , Xue, Y. , Yan, F. , Huang, D. , Wu, Y. , & Weng, Z. (2020). Polyphenol extract from superheated steam processed tea waste attenuates the oxidative damage in vivo and in vitro. Journal of Food Biochemistry, 44(1), e13096. 10.1111/JFBC.13096 31693210

[fsn34011-bib-0013] Güçlü Üstündağ, Ö. , Erşan, S. , Özcan, E. , Özan, G. , Kayra, N. , & Ekinci, F. Y. (2016). Black tea processing waste as a source of antioxidant and antimicrobial phenolic compounds. European Food Research and Technology, 242(9), 1523–1532. 10.1007/S00217-016-2653-9/TABLES/6

[fsn34011-bib-0014] Guo, S. , Kumar Awasthi, M. , Wang, Y. , & Xu, P. (2021). Current understanding in conversion and application of tea waste biomass: A review. Bioresource Technology, 338, 125530. 10.1016/J.BIORTECH.2021.125530 34271498

[fsn34011-bib-0015] Guo, Y. , Zhang, Y. , Zheng, D. , Li, M. , & Yue, J. (2020). Isolation and characterization of nanocellulose crystals via acid hydrolysis from agricultural waste‐tea stalk. International Journal of Biological Macromolecules, 163, 927–933. 10.1016/J.IJBIOMAC.2020.07.009 32640323

[fsn34011-bib-0016] Ho, K. K. H. Y. , Haufe, T. C. , Ferruzzi, M. G. , & Neilson, A. P. (2018). Production and polyphenolic composition of tea. Nutrition Today, 53(6), 268–278. 10.1097/NT.0000000000000304

[fsn34011-bib-0017] Huang, H. , Chen, J. , Chen, Y. , Xie, J. , Liu, S. , Sun, N. , Hu, X. , & Yu, Q. (2021). Modification of tea residue dietary fiber by high‐temperature cooking assisted enzymatic method: Structural, physicochemical and functional properties. LWT, 145, 111314. 10.1016/J.LWT.2021.111314

[fsn34011-bib-0018] Huang, H. , Chen, J. , Chen, Y. , Xie, J. , Xue, P. , Ao, T. , Chang, X. , Hu, X. , & Yu, Q. (2022). Metabonomics combined with 16S rRNA sequencing to elucidate the hypoglycemic effect of dietary fiber from tea residues. Food Research International, 155, 111122. 10.1016/J.FOODRES.2022.111122 35400409

[fsn34011-bib-0019] Hussain, S. , Anjali, K. P. , Hassan, S. T. , & Dwivedi, P. B. (2018). Waste tea as a novel adsorbent: A review. Applied Water Science, 8(6), 1–16. 10.1007/s13201-018-0824-5

[fsn34011-bib-0021] Ifelebuegu, A. O. , Ukpebor, J. E. , Obidiegwu, C. C. , & Kwofi, B. C. (2015). Comparative potential of black tea leaves waste to granular activated carbon in adsorption of endocrine disrupting compounds from aqueous solution. Global Journal of Environmental Science and Management, 1(3), 205–214. 10.7508/GJESM.2015.03.003

[fsn34011-bib-0022] Jiang, H. , Yu, F. , Qin, L. , Zhang, N. , Cao, Q. , Schwab, W. , Li, D. , & Song, C. (2019). Dynamic change in amino acids, catechins, alkaloids, and gallic acid in six types of tea processed from the same batch of fresh tea (*Camellia sinensis* L.) leaves. Journal of Food Composition and Analysis, 77, 28–38. 10.1016/J.JFCA.2019.01.005

[fsn34011-bib-0023] Kantar, C. , Er, Z. , Baltaş, N. , & Şaşmaz, S. (2022). Some azo compounds containing black tea processing waste Catechins As antioxidant and urease enzyme inhibitory. El‐Cezeri, 9(3), 1147–1156. 10.31202/ECJSE.1131913

[fsn34011-bib-0024] Koca, İ. , & Bostancı, Ş. (2014). Oolong Çayın Üretimi, Bileşimi ve Sağlık Üzerine Etkisi. Türk Tarım—Gıda Bilim Ve Teknoloji Dergisi, 2(3), 154–159.

[fsn34011-bib-0025] Kondo, M. , Hirano, Y. , Kita, K. , Jayanegara, A. , & Yokota, H. O. (2018). Nutritive evaluation of spent green and black tea leaf silages by in vitro gas production characteristics, ruminal degradability and post‐ruminal digestibility assessed with inhibitory activity of their tannins. Animal Science Journal, 89(12), 1656–1662. 10.1111/ASJ.13106 30318832

[fsn34011-bib-0026] Kondo, M. , Kita, K. , & Yokota, H. O. (2004). Feeding value to goats of whole‐crop oat ensiled with green tea waste. Animal Feed Science and Technology, 113(1–4), 71–81. 10.1016/J.ANIFEEDSCI.2003.10.018

[fsn34011-bib-0027] Kondo, M. , Kita, K. , & Yokota, H. O. (2006). Evaluation of fermentation characteristics and nutritive value of green tea waste ensiled with byproducts mixture for ruminants. Asian‐Australasian Journal of Animal Sciences, 19(4), 533–540. 10.5713/AJAS.2006.533

[fsn34011-bib-0028] Kondo, M. , Naoki, N. , Kazumi, K. , & Yokota, H. O. (2004). Enhanced lactic acid fermentation of silage by the addition of green tea waste. Journal of the Science of Food and Agriculture, 84(7), 728–734. 10.1002/JSFA.1726

[fsn34011-bib-0029] Kosińska, A. , & Andlauer, W. (2014). Antioxidant capacity of tea: Effect of Processing and Storage. In Processing and Impact on Antioxidants in Beverages (pp. 109–120). Academic Press. 10.1016/B978-0-12-404738-9.00012-X

[fsn34011-bib-0030] Kwon, H. L. , & Chung, M. S. (2015). Pilot‐scale subcritical solvent extraction of curcuminoids from curcuma long L. Food Chemistry, 185, 58–64. 10.1016/J.FOODCHEM.2015.03.114 25952841

[fsn34011-bib-0031] Lv, H. P. , Zhang, Y. J. , Lin, Z. , & Liang, Y. R. (2013). Processing and chemical constituents of Pu‐erh tea: A review. Food Research International, 53(2), 608–618. 10.1016/J.FOODRES.2013.02.043

[fsn34011-bib-0032] Makalesi, A. , Mortas, M. , & Awad, N. (2020). Optimization of extraction parameters by response surface methodology in handling tea extract from fibrous tea waste. European Journal of Science and Technology, 20, 672–684. 10.31590/ejosat.790454

[fsn34011-bib-0033] Marić, M. , Grassino, A. N. , Zhu, Z. , Barba, F. J. , Brnčić, M. , & Rimac Brnčić, S. (2018). An overview of the traditional and innovative approaches for pectin extraction from plant food wastes and by‐products: Ultrasound‐, microwaves‐, and enzyme‐assisted extraction. Trends in Food Science & Technology, 76, 28–37. 10.1016/J.TIFS.2018.03.022

[fsn34011-bib-0034] Martono, Y. (2010). Potency of industrial tea waste: Comparison between green and black tea industrial wastes as UV filter for sunscreen. Indonesian Journal of Cancer Chemoprevention, 1(1), 54. 10.14499/INDONESIANJCANCHEMOPREV1ISS1PP54-59

[fsn34011-bib-0035] Miao, S. , Wei, Y. , Chen, J. , & Wei, X. (2022). *Extraction methods*, *physiological activities and high value applications of tea residue and its active components: A review* . 10.1080/10408398.2022.2099343 35833478

[fsn34011-bib-0036] Mushtaq, M. , Sultana, B. , Akram, S. , Anwar, F. , Adnan, A. , & Rizvi, S. S. H. (2017). Enzyme‐Assisted Supercritical Fluid Extraction: An Alternative and Green Technology for Non‐extractable Polyphenols. Analytical and Bioanalytical Chemistry, 409, 3645–3655. 10.1007/s00216-017-0309-7 28331956

[fsn34011-bib-0037] Nadar, S. S. , Rao, P. , & Rathod, V. K. (2018). Enzyme assisted extraction of biomolecules as an approach to novel extraction technology: A review. Food Research International, 108, 309–330. 10.1016/J.FOODRES.2018.03.006 29735063

[fsn34011-bib-0038] Negi, T. , Kumar, Y. , Sirohi, R. , Singh, S. , Tarafdar, A. , Pareek, S. , Kumar Awasthi, M. , & Alok Sagar, N. (2022). Advances in bioconversion of spent tea leaves to value‐added products. Bioresource Technology, 346, 126409. 10.1016/J.BIORTECH.2021.126409 34838972

[fsn34011-bib-0039] Otağ, M. R. (2023). Siyah Çay ve Siyah Çay Atığı Örneklerinin Farklı Çözgen Konsantrasyonlarında Elde Edilen Ekstraktlarının Biyoaktif Özellikleri ve Antimikrobiyal Aktivitelerinin Belirlenmesi. Journal of Anatolian Environmental and Animal Sciences (Anadolu Çevre Ve Hayvancılık Bilimleri Dergisi), 8(1), 80–87. 10.35229/jaes.1226432

[fsn34011-bib-0040] Panneerselvam, P. , Morad, N. , & Tan, K. A. (2011). Magnetic nanoparticle (Fe3O4) impregnated onto tea waste for the removal of nickel(II) from aqueous solution. Journal of Hazardous Materials, 186(1), 160–168. 10.1016/J.JHAZMAT.2010.10.102 21146294

[fsn34011-bib-0041] Rajapaksha, S. , & Shimizu, N. (2022). Pilot‐scale extraction of polyphenols from spent black tea by semi‐continuous subcritical solvent extraction. Food Chemistry: X, 13, 100200. 10.1016/J.FOCHX.2021.100200 35498997 PMC9039883

[fsn34011-bib-0064] Roofigari Haghighat, S. , Shirinfekr, A. , Azadi Gonbad, R. , & Seraji, A. (2020). Investigation on natural color extraction from black tea waste. Journal of Medicinal Plants and By‐products, 1, 1–6. 10.22092/jmpb.2020.121745

[fsn34011-bib-0042] Salman, S. , & Özdemir, F. (2018). Beyaz Çay: Üretimi, Bileşimi ve Sağlık Üzerine Etkileri. Akademik Gıda, 16(2), 218–223. 10.24323/akademik-gida.449867

[fsn34011-bib-0043] Selahvarzi, A. , Sanjabi, M. R. , Ramezan, Y. , Mirsaeedghazi, H. , Azarikia, F. , & Abedinia, A. (2021). Evaluation of physicochemical, functional, and antimicrobial properties of a functional energy drink produced from agricultural wastes of melon seed powder and tea stalk caffeine. Journal of Food Processing and Preservation, 45(9), e15726. 10.1111/JFPP.15726

[fsn34011-bib-0044] Serdar, G. , Demir, E. , & Sökmen, M. (2017). Recycling of tea waste: Simple and effective separation of caffeine and Catechins by microwave assisted extraction (MAE). International Journal of Secondary Metabolite, 4, 78–89. 10.21448/ijsm.288226

[fsn34011-bib-0045] Sermyagina, E. , Mendoza Martinez, C. L. , Nikku, M. , & Vakkilainen, E. (2021). Spent coffee grounds and tea leaf residues: Characterization, evaluation of thermal reactivity and recovery of high‐value compounds. Biomass and Bioenergy, 150, 106141. 10.1016/J.BIOMBIOE.2021.106141

[fsn34011-bib-0046] Shalmashi, A. , Abedi, M. , Golmohammad, F. , & Eikani, M. H. (2010). Isolation of caffeine from tea waste using subcritical water extraction. Journal of Food Process Engineering, 33(4), 701–711. 10.1111/J.1745-4530.2008.00297.X

[fsn34011-bib-0063] Sökmen, M. , Demir, E. , & Alomar, S. Y. (2018). Optimization of sequential supercritical fluid extraction (SFE) of caffeine and catechins from green tea. The Journal of Supercritical Fluids, 133, 171–176. 10.1016/j.supflu.2017.09.027

[fsn34011-bib-0047] Sudheer Babu, A. , Rama Krishna, C. , Raju, S. , Vijay, K. , & Suresh Nayak, A. (2022). Evaluation of tea waste or tea residue, orange peels and pigeon pea pods for proximate composition, fodder quality and digestibility parameters. The Pharma Innovation Journal, 11(11), 1441–1445. 10.1007/978-981-13-8660-2_3

[fsn34011-bib-0048] Sui, W. , Xiao, Y. , Liu, R. , Wu, T. , & Zhang, M. (2019). Steam explosion modification on tea waste to enhance bioactive compounds' extractability and antioxidant capacity of extracts. Journal of Food Engineering, 261, 51–59. 10.1016/J.JFOODENG.2019.03.015

[fsn34011-bib-0049] Tahir, M. H. , Mubashir, T. , Hussain, M. B. , Cheng, X. , Karim, A. , Ali, N. , Jamil, M. , Khan, A. M. , & Irfan, R. M. (2021). Selective catalytic conversion of tea waste biomass into phenolic‐rich bio‐oil and subsequent extraction. Journal of Analytical and Applied Pyrolysis, 159, 105315. 10.1016/J.JAAP.2021.105315

[fsn34011-bib-0050] Tanaka, T. , & Matsuo, Y. (2020). Production mechanisms of black tea polyphenols. Chemical and Pharmaceutical Bulletin, 68(12), 1131–1142. 33268645 10.1248/cpb.c20-00295

[fsn34011-bib-0051] Tosun, İ. , & Karadeniz, B. (2005). Çay ve çay fenoliklerinin antioksidan aktivitesi. Anadolu Journal of Agricultural Sciences, 20(1), 78–83.

[fsn34011-bib-0052] Uğurlu, C. , & Güçlü Üstündağ, Ö. (2017). Recovery of L‐Theanine and Catechins from green tea waste using subcritical water extraction. In Conference: 16th European meeting on supercritical Fluids, 25–28th of April, Lisbon, Portugal.

[fsn34011-bib-0053] Vilas‐Boas, A. A. , Pintado, M. , & Oliveira, A. L. S. (2021). Natural bioactive compounds from food waste: Toxicity and safety concerns. Food, 10(7), 1564. 10.3390/FOODS10071564 PMC8304211 34359434

[fsn34011-bib-0054] Xingfei, L. , Shunshun, P. , Wenji, Z. , Lingli, S. , Qiuhua, L. , Ruohong, C. , & Shili, S. (2020). Properties of ACE inhibitory peptide prepared from protein in green tea residue and evaluation of its anti‐hypertensive activity. Process Biochemistry, 92, 277–287. 10.1016/J.PROCBIO.2020.01.021

[fsn34011-bib-0055] Xu, A. , Lai, W. , Chen, P. , Awasthi, M. K. , Chen, X. , Wang, Y. , & Xu, P. (2021). A comprehensive review on polysaccharide conjugates derived from tea leaves: Composition, structure, function and application. Trends in Food Science & Technology, 114, 83–99. 10.1016/J.TIFS.2021.05.020

[fsn34011-bib-0056] Xue, Z. , Wang, J. , Chen, Z. , Ma, Q. , Guo, Q. , Gao, X. , & Chen, H. (2018). Antioxidant, antihypertensive, and anticancer activities of the flavonoid fractions from green, oolong, and black tea infusion waste. Journal of Food Biochemistry, 42(6), e12690. 10.1111/JFBC.12690

[fsn34011-bib-0065] Yuan, L. , Wang, H. , Yin, F. , Lan, H. , & She, S. (2013). Optimization of the enzymatic extraction process of protein in tea residue by response surface methodology. Science and Technology of Food Industry, 34(07), 247–251.

[fsn34011-bib-0058] Zhang, J. , Wen, C. , Zhang, H. , Duan, Y. , & Ma, H. (2020). Recent advances in the extraction of bioactive compounds with subcritical water: A review. Trends in Food Science & Technology, 95, 183–195. 10.1016/J.TIFS.2019.11.018

[fsn34011-bib-0059] Zhao, T. , Chen, Z. , Lin, X. , Ren, Z. , Li, B. , & Zhang, Y. (2018). Preparation and characterization of microcrystalline cellulose (MCC) from tea waste. Carbohydrate Polymers, 184, 164–170. 10.1016/J.CARBPOL.2017.12.024 29352907

[fsn34011-bib-0060] Zheng, Q. , Han, C. , Zhong, Y. , Wen, R. , & Zhong, M. (2017). Effects of dietary supplementation with green tea waste on growth, digestive enzyme and lipid metabolism of juvenile hybrid tilapia, Oreochromis niloticus × O. Aureus. Fish Physiology and Biochemistry, 43(2), 361–371. 10.1007/S10695-016-0292-5/TABLES/5 27638477

